# Development and validation of a new tumor-based gene signature predicting prognosis of HBV/HCV-included resected hepatocellular carcinoma patients

**DOI:** 10.1186/s12967-019-1946-8

**Published:** 2019-06-18

**Authors:** Gui-Qi Zhu, Yi Yang, Er-Bao Chen, Biao Wang, Kun Xiao, Shi-Ming Shi, Zheng-Jun Zhou, Shao-Lai Zhou, Zheng Wang, Ying-Hong Shi, Jia Fan, Jian Zhou, Tian-Shu Liu, Zhi Dai

**Affiliations:** 1 Liver Cancer Institute, Zhongshan Hospital, Fudan University, Shanghai, 200032 China; 2 State Key Laboratory of Genetic Engineering, Fudan University, Shanghai, 200032 China; 3 Department of Medical Oncology, Zhongshan Hospital, Fudan University, Shanghai, 200032 China; 4 Department of Radiation Oncology, Zhongshan Hospital, Fudan University, Shanghai, 200032 China

**Keywords:** Hepatocellular carcinoma, Liver resection, Molecular classification, Microarray analysis

## Abstract

**Background:**

Due to the phenotypic and molecular diversity of hepatocellular carcinomas (HCC), it is still a challenge to determine patients’ prognosis. We aim to identify new prognostic markers for resected HCC patients.

**Methods:**

274 patients were retrospectively identified and samples collected from Zhongshan hospital, Fudan University. We analyzed the gene expression patterns of tumors and compared expression patterns with patient survival times. We identified a “9-gene signature” associated with survival by using the coefficient and regression formula of multivariate Cox model. This molecular signature was then validated in three patients cohorts from internal cohort (n = 69), TCGA (n = 369) and GEO dataset (n = 80).

**Results:**

We identified 9-gene signature consisting of *ZC2HC1A*, *MARCKSL1*, *PTGS1*, *CDKN2B*, *CLEC10A*, *PRDX3*, *PRKCH*, *MPEG1 and LMO2*. The 9-gene signature was used, combined with clinical parameters, to fit a multivariable Cox model to the training cohort (concordance index, ci = 0.85), which was successfully validated (ci = 0.86 for internal cohort; ci = 0.78 for in silico cohort). The signature showed improved performance compared with clinical parameters alone (ci = 0.70). Furthermore, the signature predicted patient prognosis than previous gene signatures more accurately. It was also used to stratify early-stage, HBV or HCV-infected patients into low and high-risk groups, leading to significant differences in survival in training and validation (P < 0.001).

**Conclusions:**

The 9-gene signature, in which four were upregulated (*ZC2HC1A*, *MARCKSL1*, *PTGS1*, *CDKN2B*) and five (*CLEC10A*, *PRDX3*, *PRKCH*, *MPEG1*, *LMO2*) were downregulated in HCC with poor prognosis, stratified HCC patients into low and high risk group significantly in different clinical settings, including receiving adjuvant transarterial chemoembolization and especially in early stage disease. This new signature should be validated in prospective studies to stratify patients in clinical decisions.

**Electronic supplementary material:**

The online version of this article (10.1186/s12967-019-1946-8) contains supplementary material, which is available to authorized users.

## Background

Hepatocellular carcinoma (HCC) is the sixth most common carcinoma worldwide and the third leading cause of patient’s cancer-related mortality [1, 2]. Surgical resection is one of the most effective curative treatments for HCC [3]. However, approximately 60–70% patients will still suffer from recurrence in 5 years for most patients undergoing liver resection and the clinical outcomes is still dissatisfactory [3]. It is necessary to investigate patients at high risk for poor clinical outcomes and employed effective therapies to avoid HCC recurrence. Hence, determination of novel prognostic biomarkers is vital for early diagnostic detection.

In clinical settings, prognosis assessment and decision making after surgery are based on the tumor staging systems (i.e., TNM [4], Japan Integrated Staging [5] and Barcelona Clinic liver cancer [BCLC] [6], cancer of the liver Italian program [7]). They are widely applied to guide treatment therapies or predict HCC clinical outcomes. Some studies have raised to improve the above staging system by introducing tumor characteristics, such as serum alpha fetoprotein (AFP) and pathologic features such as microvascular invasion and tumor differentiation [8, 9].

In order to optimize prognosis scoring, searching for molecular biomarkers is an expanding domain [10–12]. However, the heterogeneity of the underlying liver disease and tumor stage in HCC has challenged the use of molecular classification in different clinical settings worldwide. Currently, array-based gene expression signatures obtained from HCC tumors have been assessed in many recent studies [12–20]. These studies have clarified gene signatures predicting HCC patient recurrence or mortality; howbeit, traditional screening methods focus on few genes and lack in systematic evaluation; furthermore, the sensitivity and specificity of a single prognostic biomarker may be scarce. Hence, the problems that lack of gene signatures applied in different clinical settings still exist.

Fortunately, it is great convenient that high-throughput technologies provide real-time monitoring of different biological molecules and make it much easier to explore a great number of potential markers at once, resulting in an explosion of new biomarkers for HCC diagnostic and prognostic prediction. Recently, more than 20 various molecular signatures have been published; however, few have been externally validated [8, 14–23]. One of these externally or internally validated molecular signature is the five-gene score [16], which has been shown to be related with disease-specific survival in resected HCCs. Interestingly, the five-gene signature showed better performance than other different signatures [12, 13, 18, 22], but lacked validation in comparison to other types of curative treatments [16].

However, a more accurate and comprehensively validated method for various patient cohorts with different treatment options is still to be identified and applied in HCC clinical guidelines. In this study, we intent to identify a molecular signature to optimally predict clinical outcomes of HCC patients who underwent liver resection. We solved some important points to intensify the robustness of our molecular signatures: (1) identification in a training HCC cohort and internal validation; (2) assessing the added value of molecular signature compared with classical clinical parameters; and (3) externally validated by another cohort patients. Ultimately, we built a new nomogram combining clinical factors and gene signature to refine prognosis model assessment and overpass the dichotomy between molecular and clinical parameters.

## Materials and methods

### Patients and tissue samples

**Fig. 1:**
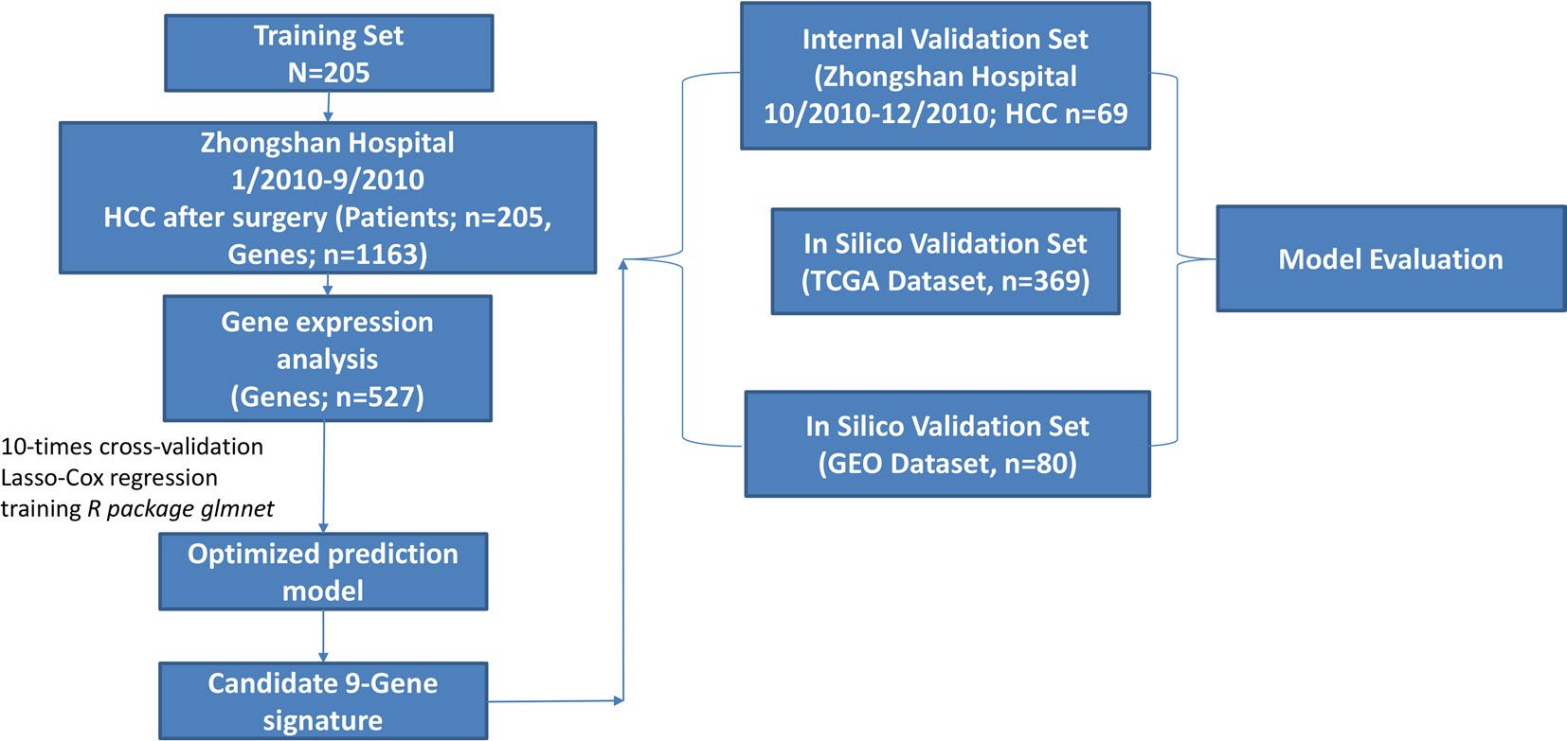
Flow chart of the study

274 archived FFPE samples were collected from Zhongshan Hospital between January 2010 and January 2011 (Fig. 1). Those samples were assigned to two phases in training, internal validation cohort in chronological order. Table 1 described the clinical parameters, which were retrospectively collected from our hospital’s medical records. Tumor stage was assigned for HCC patients whose tumors were staged before the publication of the seventh edition of the AJCC Cancer Staging Manual. The exclusion items (another 113 patients) were as follows: tumors has more than 80% of necrosis (34 patients), tumors’ RNA has poor quality or insufficient amount (30 patients), patients was performed with non-curative resection (R1 or R2 resection or extrahepatic metastasis at the time of the surgery) (25 patients), HCC patients were treated by liver transplantation (11 patients), and HCC patients were dying within the first month after liver resection due to surgical complications or decompensated cirrhosis (13 patients). The HCC diagnosis was based on established histological criteria [3]. In the terms of multiple tumors, we have taken the largest nodule of HCC into account.

**Table 1:** Patient characteristics for the discover and validation cohort

Characteristics	Training set	Internal validation set	In silico validation set	P value
No. of Patients	205	69	369	
Age (y)	53.6 ± 10.8	52.6 ± 10.8	59.4 ± 13.6	*< 0.001*
Tumor diameter (cm)	5.6 ± 4.3	4.7 ± 3.0	NA	0.263
AFP (ng/ml)	4175.1 ± 12,911.6	4022.1 ± 10,942.1	13,833.6 ± 124,798.6	0.481
*Gender*				*< 0.001*
Female	37 (18.0%)	7 (10.1%)	121 (32.8%)	
Male	168 (82.0%)	62 (89.9%)	248 (67.2%)	
*HBV*				0.731
Negative	25 (12.2%)	7 (10.1%)	22 (13.8%)	
Positive	180 (87.8%)	62 (89.9%)	137 (86.2%)	
*Adjuvant TACE*				*<* *0.001*
No	137 (66.8%)	40 (58.0%)	228 (94.6%)	
Yes	68 (33.2%)	29 (42.0%)	13 (5.4%)	
*Stage*				*<* *0.001*
I	107 (52.2%)	60 (86.9%)	172 (49.7%)	
II	74 (36.1%)	8 (11.6%)	83 (24.0%)	
III/IV	24 (11.7%)	1 (1.5%)	91 (24.9%)	
*Microvascular invasion*				*<* *0.001*
No	168 (88.9%)	67 (97.1%)	208 (66.5%)	
Yes	21 (11.1%)	2 (2.9%)	105 (33.5%)	
*Tumor differentiation*				*<* *0.001*
I/II	136 (67.3%)	55 (79.7%)	54 (14.8%)	
III/IV	66 (32.7%)	14 (20.3%)	310 (85.2%)	
*Tumor number*				*<* *0.001*
Single	171 (83.4%)	64 (92.8%)	86 (49.7%)	
Multiple	34 (16.6%)	5 (7.2%)	87 (50.3%)	

Mean + SD/N (%)

*TACE* transarterial chemoembolization, *AFP* alpha-fetoprotein

Italic P values indicate P < 0.05

### Gene expression profiling and data processing

We collected 274 HCC patient samples (including tumor and peri-tumoral tissues) from our hospital. Firstly, we removed every peri-tumoral tissue of HCC samples carefully on each FFPE sections by manual macro-dissection using the H&E stained slides as the reference under the instruction of pathologist (Sun Chun), and then extracted tumoral tissue RNA using the RNeasy FFPE kit (Qiagen, Hilden, Germany) according to the manufacturer’s protocol. NanoDrop (Thermo Scientific, Waltham, MA, USA) was used to measure the concentrations of extracted RNA samples. Samples with RNA concentrations no less than 40  ng/μL were subjected to further analysis. We used two NanoString gene panels in our study to quantify the expression of 1163 genes (Additional file 1: Table S1), which were involved in multiple signaling pathways related to HCC oncogenesis, HCC progression and/or cancer immunology. We measured these 1163 genes rather than entire gene profile because the aim of study was to focus on analyzing those genes, which had been proved to be relevant to HCC. 200 ng RNA were hybridized overnight at 65 °C with probes according to the manufacturer’s protocol, followed by digital barcode counting using NanoString nCounter Digital Analyzer (NanoString Technologies, Seattle, WA, USA). The logarithm of raw counts was normalized by the expression of positive controls and then submitted to further statistical analysis.

Gene expression profiling of 274 HCC patients were detected. We processed the gene expression data using ‘glmnet’ package of R software (version 3.3.1, R Foundation for Statistical Computing Vienna, Austria). The following steps for this well-defined process: firstly, importing the ‘raw’ data in.CEL format and the associated clinical survival information; secondly, summarizing the gene expression values for each probe set; the last step included background adjustment and z-score normalization.

### Identification of the prognostic gene signature

Firstly, we constructed the gene signature from the gene expression matrix of 205 HCC patients. The ‘scipy’ package (scipy.stats.pearsonr) of Python was used to determine whether gene expression correlated with overall survival (OS). 527 genes with a *P* value ≤ 0.05 from 1163 genes obtained from 205 HCC patients gene expression profiles were used for the subsequent regression model analysis. In general, we used Lasso (shrinkage and selection method for linear regression) to perform the regression analysis in the data matrix (274 * 470). Additionally, we used the penalized regression model with LASSO penalty to achieve shrinkage and variable selection simultaneously, and the optimal values of the penalty parameter alpha were determined through 10-times cross-validations. Based on the optimal alpha value, nine prognostic genes with corresponding coefficients were screened out of 527 genes based on gene expression profiling and OS data (Fig. 1). The Predicted OS information for each patient was then calculated based on the expression level of each prognostic gene and its corresponding coefficient. Then, R-squared values were calculated to evaluate concordance between predicted OS and real OS in two optimized algorithms, Lasso and LassoLars. Prognostic models including the following parameters were compared: the identified gene signature alone, clinical parameters alone, and the combination of clinical parameters and the identified gene signature.

### Experimental and in silico signature validation

We used three validation cohorts (one internal and two in silico datasets) of HCC in the prognostic study (Fig. 1): internal validation set (n = 69, HCC patients undergoing surgery from October 2010 to January 2010); and two independent cohorts in silico, TCGA set (n = 369, HCC patients undergoing surgery) and HCC GEO dataset (n = 80, GSE10143) [24].

### End points

We designed the study following some recommendations for prognostic cancer biomarkers included in the Reporting Recommendations for Tumor Marker Prognostic Studies statement (REMARK) [7, 25] and the European Association for the Study of the Liver/European Organization for Research and Treatment of Cancer guidelines (EASLORTC) [25, 26]. Three steps were endorsed by these guidelines for HCC prognostic biomarkers: (1) identifying biomarkers need to follow a mode of training and validation form; (2) independent prognostic value of biomarkers should retain when meeting with known clinical or pathological parameters; and (3) biomarkers need to be validated in any in silico datasets of independent patients.

We followed patients and screened HCC recurrence by serum level of AFP and computed tomography scan or liver magnetic resonance imaging after liver resection. We determined the primary end point in our study was overall survival (OS), defined by the interval between the date of surgery and all-cause death (including tumor-specific death and other cause deaths) or the last follow-up. The last recorded follow-up was July 2015. The disease-free survival (DFS) was also assessed, which was determined by analyzing patients death and censoring patients who suffered from first tumor relapse (When the level of postoperative serum AFP was > 20 ng/mL and new focus appeared in the ultrasonic/abdominal computed tomography during follow up, we considered that they had tumor recurrence), tumor-related death or progression. Specifically, tumor-related death (which was defined when patient’s death occurred in HCC involving > 50% of the liver, HCC with extensive tumor portal thrombosis, or extrahepatic metastasis). Additionally, in order to limit the background noise due to the occurrence of a second independent HCC, we censored survival at 5 years after the initial resection surgery.

### Statistical analysis

We estimated the survival curves by the Kaplan–Meier analysis and compared by log-rank tests. Differences between the training and validation cohorts were evaluated by the Mann–Whitney U test for continuous variables and the Chi squared test for categorical variables. We performed descriptive analyses and the described statistical tests using SPSS statistical software, version 23 (IBM Corporation, New York, USA). A statistical framework was constructed to identify potential gene signatures and corresponding prognostic models to optimally predict the primary and secondary endpoints. In order to evaluate the prognostic performance of our developed models, the concordance index (ci) was calculated (31). While ci = 0.5 was obtained for a non-informative model, ci = 1.0 represents a perfectly predicting model. To compare the performance between nested multivariable Cox models, the likelihood ratio test was applied. The model was validated using the independent validation cohort in silico (TCGA and GEO datasets). The 95% confidence interval (CI) of the ci was estimated from 1000 bootstrap samples of the training and validation cohort. Finally, the validation was declared successful if the 95% CI did not contain 0.5. In addition, interaction and stratified analyses were conducted according to age (< 60 and > 60 years), gender (male and female), HBV status (positive and negative), receiving adjuvant transarterial chemoembolization (TACE) (yes and no), AFP (< 200 and > 200 ng/ml), tumor parameters (tumor diameters < 5 and > 5 cm, tumor differentiation (I/II and III/IV), tumor numbers (single and multiple), and TNM stage (I/II and III/IV). The framework to determine gene signatures and corresponding prognostic models was implemented in R Statistics version 3.3.2 (R Foundation for Statistical Computing, Vienna, Austria (32, 33). For all analyses, two-sided tests were performed and P-values below 0.05 were considered statistically significant. We included the three most significant clinical, pathological, and molecular parameters in the all cohorts of HCC patients to build a composite prognostic model by using the R package: rms (http://www.R-project.org/).

## Results

### Patient cohorts

In this retrospective study, a training cohort of 205 patients and an independent, monocenter internal validation cohort of 69 patients and in silico validation cohorts of 369 patients with resected HCC were available for the development of a gene signature to predict the clinical endpoints of DFS and OS. Patient data, treatment parameters, and tumor characteristics of both patient cohorts are summarized in Table 1. The entire cohort was mostly male (74%) with a mean age of greater than 50 years and with the following tumor parameters: single tumor number (50%), tumor diameter (5.3 ± 4.0 cm), tumor differentiation (I/II: 38.5%; III/IV: 61.4%), TNM stage (I: 59.1%; II: 28.7%; III/IV: 18.0%) or receiving adjuvant TACE (yes: 35.4%; no: 64.6%). In addition, HBV DNA-positive patients in the entire cohort was 87.8% in the training set vs. 89.9% in the internal validation set vs. 37.1% in the TCGA and GEO dataset. A first cohort of 205 patients treated by liver surgery was used at Zhongshan Hospital of Fudan University to identify a gene signature and an internal, and two in silico validation cohorts of 69, 369, and 80 patients in the TCGA or GEO dataset (Table 1 and Fig. 1).

### A nine-gene signature associated with prognosis in resected HCC training and validation cohorts

**Fig. 2:**
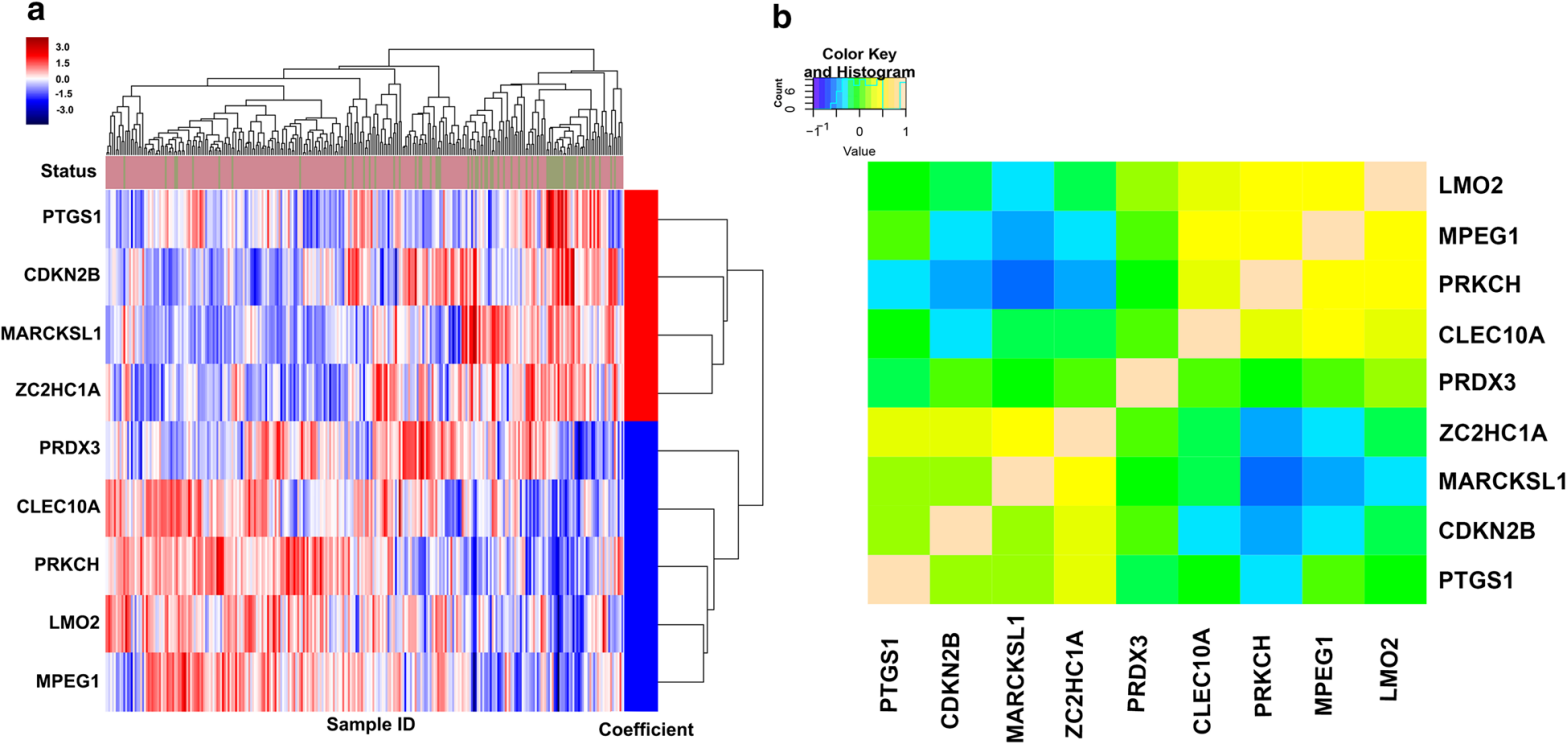
The heatmap of selected 9-gene signature and the correlation map of gene expression for each genes. **a** The selected corresponding nine genes of heatmap by overall survival. **b **The correlation map of gene expression for each nine genes

In the training cohort (205 HCC patients at Zhongshan Hospital), we found a panel of nine genes, including Myristoylated alanine-rich C kinase substrate like-1 (MARCKSL1), zinc finger C2HC-type containing 1A (ZC2HC1A), prostaglandin-endoperoxide synthase 1 (PTGS1), cyclin dependent kinase inhibitor 2B (CDKN2B), C-type lectin domain family 10 member A (CLEC10A), Peroxiredoxin2 (PRDX2), Protein kinase C eta (PRKCH), Macrophage expressed 1 (MPEG1), LIM domain only 2 (LMO2), which showed the strongest prognostic relevance (Fig. 2b). Among these genes, four were upregulated (ZC2HC1A, MARCKSL1, PTGS1, CDKN2B) and five (CLEC10A, PRDX3, PRKCH, MPEG1, LMO2) were downregulated in HCC with poor prognosis (Additional file 2: Table S2). We then constructed a nine-gene signature using the coefficient formulas of the multivariate Cox model from the training population. The formula as follows: 0.142772 * ZC2HC1A + 0.109389 * MARCKSL1 + 0.097517 * PTGS1 + 0.001941 * CDKN2B-0.00375 * CLEC10A-0.03609 * PRDX3 − 0.06777 * PRKCH-0.07912 * MPEG1 − 0.14638 * LMO2. The corresponding nine genes of heatmap are presented in Fig. 2a. Finally, we validated this nine-gene signature and clinical parameters selected by the multivariable Cox model in the independent internal or in silico validation cohorts. The dichotomized nine-gene score by median value was associated with OS significantly in the training and validation cohorts (P < 0.01, Additional file 3: Figures S1IA–C). In addition, nine genes each dichotomizing into low/high risk (according to median expression value) were significantly associated with OS in the training cohort, except for PRDX3 (P = 0.149, see Additional file 3: Figure S1II). In order to estimate the accuracy of the nine-gene signature to predict DFS and OS, the area under the curve (AUC) of the nine-gene signature in the training cohort was calculated, which we then computed in the internal or in silico validation populations (Additional file 3: Figures S1ID, E). The AUC was also calculated at different time points of DFS and OS (1-year, 3-year, and 5-year, respectively); the summary measure of the AUC all reached over 0.70, showing good consistency performance of the nine-gene signature regardless of different time points for OS and DFS (Additional file 4: Figure S2I).

Among all 643 resected HCC patients, nine-gene signature classified 321 patients into the poor prognosis group. This molecular poor prognosis group was associated with previously important clinical parameters (tumor size and stage) significantly and pathological (microvascular invasion and tumor differentiation) characteristics (Additional file 5: Table S3). In contrast, our gene signature was not related to age, gender, non-HBV liver disease, tumor number or receiving adjuvant TACE.

### Inclusion of clinical parameters to the nine-gene HCC signature

We next to evaluate the independent predictive prognostic value of the nine-gene signature. The nine-gene signature was related to OS irrespective of some clinical and pathological factors, including staging, for the training and validation cohorts (Table 2). As our training cohort reflecting the diversity of HCC in term of stage, tumor etiology, or receiving adjuvant therapy, we evaluated the predictive performance of our nine-gene signature in each condition (Additional file 4: Figure S2II). Interestingly, our nine-gene signature was associated with OS in each subgroup significantly, regardless of age, gender, serum levels of AFP, tumor number, tumor size, tumor differentiation, except for tumor stage III/IV and the presence of microvascular invasion (Additional file 4: Figure S2II). Similarly, the Kaplan–Meier analyses showed our molecular signature conferred significant survival benefits in terms of tumor characteristics, such as tumor stage (stage I or II; both P < 0.001), tumor differentiation (P < 0.001), and clinical parameter: presence of microvascular invasion (P < 0.0001; see Additional file 6: Figure S3I). Interestingly, to explore the prognostic value of the nine-gene signature for other treatment options, receiving adjuvant TACE after curative resection accounted for 35.4% in the training and validation cohorts. Stratified analyses showed that the nine-gene signature also enabled to predict prognosis for HCC patients receiving adjuvant TACE (HR = 3.2, P < 0.001; Additional file 4: Figure S2II, Additional file 6: Figure S3I).

**Table 2:** Univariable and multivariable Cox regression of overall survival in training and validation cohort

	Statistics	Univariate analysis		Multivariate analysis	
		HR (95% CI)	P value	HR (95% CI)	P value
*Age*					
< 65	234 (85.4%)	1.0			
> 65	40 (14.6%)	0.8 (0.4, 1.6)	0.458		
*Gender*					
Female	44 (16.1%)	1.0			
Male	230 (83.9%)	1.5 (0.7, 3.1)	0.291		
*Tumor number*					
Single	235 (85.8%)	1.0			
Multiple	39 (14.2%)	1.3 (0.7, 2.4)	0.486		
*Stage*					
I	167 (60.9%)	1.0			
II	82 (29.9%)	2.0 (1.1, 3.7)	*0.026*		
III/IV	25 (9.1%)	3.6 (1.7, 7.6)	*<* *0.001*		
*Adjuvant TACE*					
No	177 (64.6%)	1.0			
Yes	97 (35.4%)	1.8 (1.1, 3.0)	*0.015*		
*HBV*					
Negative	32 (11.7%)	1.0			
Positive	242 (88.3%)	1.7 (0.7, 4.2)	0.269		
Tumor diameter	5.4 + 4.1	1.2 (1.1, 1.2)	*<* *0.001*	1.36 (1.24, 1.48)	*<* *0.0001*
AFP	4143.4 + 12,507.8	1.0 (1.0, 1.0)	0.071		
*Microvascular invasion*					
No	235 (91.1%)	1.0			
Yes	23 (8.9%)	3.0 (1.5, 6.0)	*0.002*		
*Tumor differentiation*					
I/II	191 (70.5%)	1.0			
III/IV	80 (29.5%)	2.3 (1.4, 3.8)	*0.001*	11.27 (1.85, 68.53)	*0.008*
9-gene signature	− 0.1 + 0.4	7.5 (5.0, 11.2)	*<* *0.001*	2.94 (1.24, 6.99)	*0.014*

*TACE* transarterial chemoembolization, *AFP* alpha-fetoprotein

Italic P values indicate P < 0.05

Specifically, in our training and TCGA validation cohorts, 504 (81.3%) of HCC patients was classified into stage I/II disease (Table 1). Therefore, we measured predictive performance of our nine-gene signature in HCC patients with early (Stages I and II) disease. Firstly, we indicated that HCC patients in stage I (n = 339) and II (n = 165) (from training and validation populations) showed a wide range of survival times, from a few months to more than 10 years. The nine-gene signature accurately predicted OS of these patients in the Kaplan–Meier analyses (stage I, P = 0.0032, HR = 2.1; stage II, P < 0.0001, HR = 6.1) (Additional file 4: Figure S2II and Additional file 7: Figure S4II). The nine-gene signature was also evaluated in different subgroups of HCC patients (Additional file 4: Figure S2II). Interestingly, it did not predict survival of stage III or IV HCC patients (P = 0.1; Additional file 7: Figure S4II) and the interaction analyses showed that significant differences only existed in Stage I, II, and III/IV patients (P = 0.03; Additional file 4: Figure S2II). Therefore, the nine-gene signature may allow a reliable prediction of survival in early-stage HCC patients.

Then, we compared the AUC of the nine-gene signature with clinical parameters only in the entire cohort, showing that the nine-gene signature outperformed clinical parameters only (AUC: 0.842 vs 0.751, P < 0.001, Additional file 3: Figure S1IF). For the training cohort, it was shown that the established clinical parameters of tumor diameter (< 5 cm or > 5 cm) and tumor differentiation (I/II or III/IV) significantly correlated with OS or DFS. For short term OS (Table 3), using only these two parameters resulted in a lower performance (Training: ci = 0.70 (0.46, 0.94), internal validation: ci = 0.68 (0.21, 0.98); in silico validation ci = 0.54 (0.42, 0.78)) compared with the nine-gene signature (training: ci = 0.79 (0.62, 0.97); internal validation ci = 0.77 (0.55, 0.99); in silico validation ci = 0.65 (0.57, 0.99)). Finally, both the clinical parameters of tumor diameter (< 5 cm vs. > 5 cm) and tumor differentiation (I/II vs. III/IV) as well as the nine-gene signature, increased the training ci to 0.85 (0.74, 0.99), the internal validation ci to 0.86 (0.58, 1.13), and the in silico validation to 0.78 (0.61, 0.98). While in training, the clinical Cox model was significantly improved by adding clinical parameters (P = 0.001), adding the nine-gene signature to the clinical parameters resulted only in a small improvement (P = 0.082).

**Table 3:** Multivariable Cox regression of short-term overall survival

Parameter	HR (95% CI)	P value	ci training (95% CI)	ci internal validation (95% CI)	ci in silico validation (95% CI)
9-gene signature	3.37 (1.53, 7.49)	< 0.0001	0.79 (0.62, 0.97)	0.77 (0.55, 0.99)	0.65 (0.57, 0.99)
*Clinical parameters*					
Tumor diameter	1.10 (0.98, 1.16)	0.092			
Tumor differentiation	1.84 (1.55, 6.09)	0.002	0.70 (0.46, 0.94)	0.68 (0.21, 0.98)	0.54 (0.42, 0.78)
*9-gene signature and clinical parameters*					
9-gene signature	15.38 (5.02, 47.71)	< 0.0001			
Tumor diameter	1.07 (1.01, 1.20)	0.003			
Tumor differentiation	1.39 (1.10, 3.30)	< 0.01	0.85 (0.74, 0.99)	0.86 (0.58, 1.13)	0.78 (0.61, 0.98)

Improvement of combined model compared to			dLL	Degrees of freedom	P value
9-Gene signature only			7.24	2	< 0.001
Clinical parameters only			23.41	2	0.082

Three multivariable Cox regression models were built using the training cohort: a model consisting of only the 9-gene signature (top), a model consisting only of the clinical tumor diameter and tumor differentiation, and a model combining both the 9-gene signature and clinical parameters (bottom). HRs are given with their 95% CIs and the corresponding P values. For each model, the concordance index (ci) is given for the training and internal validation cohort as well as for the patients of the or in silico validation cohort. Its 95% CI is determined from 1000 bootstrap samples of the respective cohort. The improvement of the combined model, including the 9-gene signature and the clinical parameters, compared with the 9-gene signature and clinical parameters alone is shown (bottom) based on the difference in log-likelihood (dLL)

For long term survival (Table 4), using only these two parameters resulted in lower performance (training: ci = 0.69 (0.49, 0.89), internal validation: ci = 0.73 (0.24, 1.22); in silico validation ci = 0.56 (0.23, 0.89)) compared with the nine-gene signature [training: ci = 0.78 (0.61, 0.95); internal validation ci = 0.75 (0.52, 0.96); in silico validation ci = 0.61 (0.50, 0.84)]. Finally, both the clinical parameters of tumor diameter (< 5 cm vs. > 5 cm) and tumor differentiation (I/II vs. III/IV) as well as the nine-gene signature, increased the training ci to 0.81 (0.71, 0.91), the internal validation ci to 0.86 (0.53, 1.19), and in silico validation ci to 0.74 (0.58, 0.98). In terms of primary endpoint, DFS was considered. The nine-gene signature for resected HCC combined with the clinical features of tumor diameter and tumor differentiation was trained and validated for DFS, yielding the same trending results with OS (Table 5).

**Table 4:** Multivariable Cox regression of long-term overall survival

Parameter	HR (95% CI)	P value	ci training (95% CI)	ci internal validation (95% CI)	ci in silico validation (95% CI)
9-gene signature	5.36 (3.12, 9.21)	< 0.0001	0.78 (0.61, 0.95)	0.75 (0.52, 0.96)	0.61 (0.50, 0.84)
*Clinical parameters*					
Tumor diameter	1.12 (1.06, 1.18)	0.0001			
Tumor differentiation	1.62 (0.75, 3.51)	0.222	0.69 (0.49, 0.89)	0.73 (0.24, 1.22)	0.56 (0.23, 0.89)
*9-gene signature and clinical parameters*					
9-gene signature	5.02 (2.70, 9.36)	< 0.0001			
Tumor diameter	1.11 (1.03, 1.17)	0.002			
Tumor differentiation	1.40 (1.03, 2.55)	0.003	0.81 (0.71, 0.91)	0.86 (0.53, 1.19)	0.74 (0.58, 0.98)

Improvement of combined model compared to			dLL	Degrees of freedom	P value
9-Gene signature only			11.21	2	< 0.001
Clinical parameters only			92.11	2	0.003

Three multivariable Cox regression models were built using the training cohort: a model consisting of only the 9-gene signature (top), a model consisting only of the clinical tumor diameter and tumor differentiation, and a model combining both the 9-gene signature and clinical parameters (bottom). HRs are given with their 95% CIs and the corresponding P values. For each model, the concordance index (ci) is given for the training and internal validation cohort as well as for the patients of the or in silico validation cohort. Its 95% CI is determined from 1000 bootstrap samples of the respective cohort. The improvement of the combined model, including the 9-gene signature and the clinical parameters, compared with the 9-gene signature and clinical parameters alone is shown (bottom) based on the difference in log-likelihood (dLL)

**Table 5:** Multivariable Cox regression of disease-free survival

Parameter	HR (95% CI)	P value	ci training (95% CI)	ci internal validation (95% CI)	ci in silico validation (95% CI)
9-gene signature	4.44 (2.36, 8.33)	< 0.0001	0.70 (0.58, 0.82)	0.74 (0.53, 0.95)	0.65 (0.55, 0.83)
*Clinical parameters*					
Tumor diameter	1.13 (1.06, 1.20)	0.0001			
Tumor differentiation	1.58 (0.89, 2.80)	0.115	0.64 (0.50, 0.78)	0.67 (0.24, 0.99)	0.57 (0.41, 0.73)
*9-gene signature and clinical parameters*					
9-gene signature	3.95 (0.68, 7.45)	< 0.0001			
Tumor diameter	1.08 (1.01, 1.12)	0.010			
Tumor differentiation	1.29 (0.68, 2.50)	0.422	0.79 (0.55, 1.03)	0.83 (0.57, 1.36)	0.70 (0.58, 0.92)

Improvement of combined model compared to			dLL	Degrees of freedom	P value
9-Gene signature only			10.53	2	< 0.001
Clinical parameters only			83.21	3	0.01

Three multivariable Cox regression models were built using the training cohort: a model consisting of only the 9-gene signature (top), a model consisting only of the clinical tumor diameter and tumor differentiation, and a model combining both the 9-gene signature and clinical parameters (bottom). HRs are given with their 95% CIs and the corresponding P values. For each model, the concordance index (ci) is given for the training and internal validation cohort as well as for the patients of the or in silico validation cohort. Its 95% CI is determined from 1000 bootstrap samples of the respective cohort. The improvement of the combined model, including the 9-gene signature and the clinical parameters, compared with the 9-gene signature and clinical parameters alone is shown (bottom) based on the difference in log-likelihood (dLL)

**Fig. 3:**
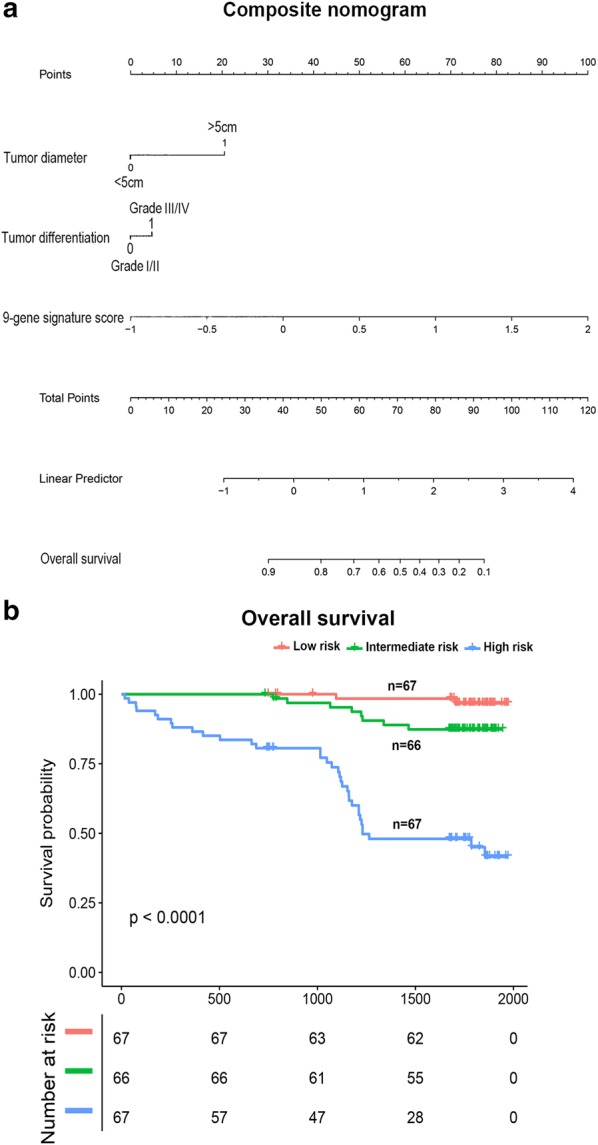
Development and Kaplan–Meier analyses of a composite nomogram to predict survival. The clinic-molecular nomogram integrated the 9-gene signature. Each component gives points and the sum of the points calculated a linear predictor and overall survival (**a**). The whole population was divided in 3 subgroups according to the total number of points given by the nomogram: patients at low risk, intermediate risk, and high risk of survival (**b**)

Finally, our established composite nomogram included tumor diameter, tumor differentiation, and nine-gene signature. The contribution of each parameters to predict OS at 5 years was showed in the nomogram (Fig. 3a). The combined scoring model divided HCC patients into 33rd and 66th percentiles accurately according to low, intermediate, and high risk categories (Fig. 3b).

### Nine-gene signature predicts prognosis of HBV-DNA positive HCC patients

Because HCC patients were predominantly HBV infected (86%) in our training or validation cohort, we further measured the performance of identified nine-gene signature and clinical parameters in patients with HBV infected for OS (Additional file 8: Table S4).

First, we evaluated the clinical benefits of the nine-gene signature only in patients with HBV-DNA positive tumors (Additional file 7: Figure S4I). The nine-gene signature predicted 3-year DFS (P < 0.019), DFS (P < 0.0001), 3-year OS (P < 0.0001), and OS (P < 0.0001) in the training and internal validation set (Additional file 7: Figure S4I). In addition, for the in silico validation of the TCGA dataset, the nine-gene signature was also significantly associated with 3-year OS (P = 0.02) and 5-year OS (P = 0.021).

Using only these two parameters resulted in a lower performance (training: ci = 0.68 (0.31, 1.04), validation: ci = 0.69 (0.49, 0.89)) compared with the nine-gene signature [training: ci = 0.77 (0.65, 0.89); validation ci = 0.73 (0.55, 0.85)]. Finally, both the clinical parameters of tumor diameter and tumor differentiation, as well as the nine-gene signature, increased the training ci to 0.85 (0.75, 0.95) and validation ci to 0.82 (0.68, 0.96). The clinical Cox model was significantly improved by adding clinical parameters (P = 0.01) and adding the nine-gene signature to the clinical parameters also resulted in significant improvement (P = 0.001).

### In silico validation of nine-gene signature in microarray-based aHCC and comparison with other molecular biomarkers

The nine-gene signature was further evaluated in another cohort of 80 resected HCCs from HCC Genomic Consortium (GSE10143) [24]. In this 80 HCC patient series, 71% of patients had hepatitis C. Additional file 7: Figure S4I showed that the nine-gene signature also enabled to predict OS (P < 0.0001) for patients primarily with HCV affection.

As for many gene expression signatures derived from tumors, the prognostic value of the five-gene signature outperformed previous molecular signatures [16], such as the proliferative signature [27], metastasis signature [22], and the hypoxia signature [18]. However, in this HCC cohort patients, the nine-gene signature (P = 0.0016) was better associated with OS than the five-gene signature (our 9-gene signature are not comprised these five genes; nonsignificant, P = 0.064; Additional file 6: Figure S3IIA-B). In addition, our bivariate analysis showed the nine-gene signature’s superiority to predict patients prognosis in the setting of surgically resected HCC patients [24] (n = 80, GSE10143, Additional file 9: Table S5). Altogether, these findings revealed that the superiority of our nine-gene signature to predict resected HCC patient prognosis when compared with other molecular biomarkers.

## Discussion

In our study, we proposed the nine-gene signature associated with HCC prognosis closely after liver surgery or adjuvant TACE. This 9-gene signature showed improved prognostic accuracy when compared with the previous five-gene signature [16]. In the Nault et al. study, an established five-gene signature (P < 0.00003 for overall survival) was shown to be better associated with survival than other proliferative [27], metastasis [22], and hypoxia signatures [18]. In addition, there’s another advantage for our gene signature to confer a continuous evaluation of OS rates for individual HCC patients. We presented to challenge the opposition between classical molecular and clinical or pathological biomarkers. Considering the above aim, we combined our nine-gene signature with clinical parameters in a new nomogram to refine model’s predictive performance.

Our nine identified genes contained genes *ZC2HC1A*, *MARCKSL1*, *PTGS1*, *CDKN2B*, *CLEC10A*, *PRDX3*, *PRKCH*, *MPEG1*, and *LMO2*. MARCKSL1 is a membrane-bound protein that is associated with cell spreading, integrin activation, and exocytosis [28]. In a prospective clinical study including 305 cancer patients [28], MARCKSL1 has a strong prognostic value in lymph node-negative cancer patients, especially in those with high proliferation. The final Cox model predicted that a high expression of MARCKSL1 was related to lower HCC survival, which may be due to its role in activating cell spreading and growth. However, to date, very little is known about ZC2HC1A and PTGS1 and their roles in cancer. PTGS1 (also known as COX1), is a critical lipid metabolism molecular protein, and has been shown to be a pro-inflammatory mediator associated with an increased risk of colon cancer [29]. CDKN2B (also named INK4B), a key cell cycle inhibitor, is related to the cell cycle and TGF-beta signaling pathway in cancer [30, 31]. A recent study [32] showed that loss of STAT5 from hepatocytes in liver tissue lead to enhanced proliferation, which was linked to reduced levels of cell cycle inhibitors p15 (INK4B) and p21 (CIP1). In a clinical study, a genetic variant of CDKN2B had an increased risk of cancer susceptibility [33]. CLEC10A induces both the production and secretion of interleukin (IL)-10 [34], while decreasing the levels of TGF-β. IL-10 triggers anticancer immunity in the tumor microenvironment [35]. Hence, downregulated expression of CLEC10A is associated with poor prognosis in HCC, which is in line with our findings.

PRDX2 is a member of the peroxiredoxin family of antioxidant enzymes. One previous study has showed that PRDX2 acted as a cell type-dependent role in tumorigenesis [36]. The new target of miR-122a has been proved to be PRDX2 [37], revealing downregulated in HCC, which is similar to our results. PRKCH is one of the members of the protein kinase C family [38]. Several studies have showed that the role of PRKCH played an important part in apoptosis and anti-apoptosis [39, 40]. The low expression of PRKCH could inhibit the growth of breast cancer cells, which is conversely upregulated expression in breast cancer. In addition, PRKCH could contribute to resistance against the breast cancer cell death by inhibiting JNK activity [41]. Aberrant signal transduction via protein kinases such as PKC may occur during liver cancer development [42]. MPEG1 is overexpressed in several human cancer tissues, including pancreas, breast, lung, liver, and thyroid [43]. Some studies revealed that the depletion of MPEG1 could impact cell mitosis [44]. A lack of MPEG1 disturbs centrosome duplication, and induces chromosome misalignment and mis-segregation [44]. Additionally, depletion of MPEG1 restrains HCC cells uncontrollable growth [42, 45]. LMO2 is a member of a transcription factor family of proteins and is a determinant of vascular development in the zebrafish because of an effect on embryonic angiogenesis, which seems to be on endothelial cell migration, rather than proliferation [46, 47].

The identified nine-gene signature showed good prognostic ability for endpoint DFS or OS in the validation cohort (ci = 0.70 for DFS, ci = 0.78 for OS). The predictive performance of the signature was testified in different HCC patient subgroups (Additional file 7: Figure S4II). This consistency across different subgroups of patients reveals that the nine-gene signature determining disease progression and survival are conserved regardless of HCC heterogeneity. This is notable, since HCC is known to be derived from various cell types, including hepatocytes, adult stem or progenitor cells [48] and is led by several etiologies. When combined with the clinical parameters of tumor diameter and tumor differentiation, its performance could be further improved (ci = 0.79 for DFS; ci = 0.85 for OS). This indicates that the combination of well-established clinical parameters and prognostic biomarkers may lead to a more accurate prognosis than each of them alone. The model including only clinical parameters showed the lowest validation performance (ci = 0.68 for OS; ci = 0.67 for DFS). In the Cox model combining clinical parameters with the nine-gene signature, most signature genes were significantly associated with patient survival. The final Cox model showed better performance in the training cohort (ci = 0.83) than in the validation cohort (ci = 0.77 for internal validation; ci = 0.65 for in silico validation). This difference is expected, since the final Cox model is adjusted to the training cohort and potential overfitting might occur. In addition, the validation of the proposed nine-gene signature might be impeded by the significant differences between both patient cohorts. In the validation cohorts, patients were clinically characterized by a higher percentage of HCV-infection, higher tumor numbers, and higher tumor stage. On the other hand, the in silico validation cohort had a higher percentage of unfavorable tumor differentiation (III/IV: 84.9%) than the training cohort (III/IV: 32.6%). These negative prognostic factors outbalanced the positive ones, resulting in differences in outcomes.

Our validation HCC cohort is characterized by different etiologies (e.g., hepatitis C, and hepatitis B virus) and by various tumor stages from early to advanced HCC. In comparison to other studies that mainly focused on HBV-related HCC, we validated our gene signature in another two in silico HCC cohorts, mainly related to HCV infection in Western patients and HBV infection in Eastern patients, all revealing its good predictive performance in different clinical settings. In all, we validated our nine-gene signature in 723 HCC patients undergoing liver resection worldwide and in different settings (Additional file 6: Figure S3I).

In the future, the values of our nine-gene signature also need to be validated in clinical guidelines. Firstly, we could use the nine-gene signature to stratify the risk of HCC patient survival before the decision for liver surgery is made. Particularly, the 9-gene signature, in the presence of MVI and advanced stage HCC, could identify HCC patients with a good prognosis that would benefit from therapy, and patients with a poor prognosis who could avoid unnecessary surgery [49, 50]. Evidently, we need to validate the gene signature in prospective studies and in other kinds of curative therapies. Even though the finite treatment options after curative resection in our routine clinical practice, the nine-gene signature could also classify death risk after liver surgery combined with adjuvant TACE (Additional file 7: Figures S4I, II).

## Conclusions

Overall, we have proposed a nine-gene signature in HBV/HCV-included HCC patients who underwent resection in one independent hospital and further validated its predictive accuracy in three cohort populations. In addition, our nine-gene signature obeys REMARK guidelines and the EASLORTC for a prognostic biomarker in HCC patients undergoing curative resection [25, 26]. However, we still need to evaluate the nine-gene signature and validate its application in clinical and therapeutic decision making for HCC patients (Additional file 6: Figure S3II).

## Additional files

**Additional file 1: Table S1.** Name list of the genes tested by Nanostring in our study for further analysis.

**Additional file 2: Table S2.** The coefficient and regulation of 9-gene signature.

**Additional file 3: Figure S1.** The area under the curve related to overall survival, the Kaplan–Meier analyses according to 9-gene signature (Panel I) and each nine genes (Panel II) in training and validation cohorts. Panel I: A: training set; B: internal validation set; C: in silico validation set; D. training set; E. validation set; F. entire set. Panel II: The genes were divided by median expression.

**Additional file 4: Figure S2.** The time-dependent area under the curve related to overall survival, disease-free survival at different years (Panel I; 1-year, 3-year and 5-year) and the subgroup analysis for 9-gene signature in different clinical characteristics (Panel II).

**Additional file 5: Table S3.** Correlation between 9-gene signature and clinicopathologic characteristics from training and validation cohort.

**Additional file 6: Figure S3.** The Kaplan–Meier analyses according to 9-gene signature in different subgroups (Panel I) and the comparison of 9-gene signature with other molecular score for Kaplan–Meier analyses analysis. Panel II: A: 5-gene signature; B: 9-gene signature.

**Additional file 7: Figure S4.** The Kaplan–Meier analyses for HBV-related (Panel I) and different stage HCC patients (Panel II) according to 9-gene signature in training and validation cohort in terms of overall survival and disease-free survival. Panel II: A: stage I HCC patients; B: stage II HCC patients; C. stage III/IV HCC patients.

**Additional file 8: Table S4.** Multivariable Cox regression of overall survival for patients with HBV-DNA positive tumors.

**Additional file 9: Table S5.** Comparison of the 9-gene signature and the 5-genes signature to predict overall survival using bivariate analysis in GEO set of patients (n=80).

## Data Availability

Two independent cohorts, TCGA set (n = 369, HCC patients undergoing surgery) was obtained from https://cancergenome.nih.gov/, and GSE10143 was downloaded from GEO (http://www.ncbi.nlm.nih.gov/geo/). Our expression profiling arrays were analyzed by the ‘scipy’ package (scipy.stats.pearsonr) of Python to determine whether gene expression correlated with prognosis.

## Abbreviations

HCC: hepatocellular carcinoma
AFP: α-fetoprotein
REMARK: Reporting Recommendations for Tumor Marker Prognostic Studies
DFS: disease free survival
OS: overall survival
CI: confidence interval
HR: hazard risk
TACE: transarterial chemoembolization
TNM: tumor-node-metastasis
BCLC: Barcelona Clinic Liver cancer
ci: concordance index
HBV: hepatitis B virus
MARCKSL1: myristoylated alanine-rich C; kinase substrate like-1
ZC2HC1A: zinc finger C2HC-type containing 1A
PTGS1: prostaglandin-endoperoxide synthase 1
CDKN2B: cyclin dependent kinase inhibitor 2B
CLEC10A: C-type lectin domain family 10 member A
PRDX2: peroxiredoxin2
PRKCH: protein kinase C eta
MPEG1: macrophage expressed 1
LMO2: LIM domain only 2
