# Supplementary material for: Development and validation of a new tumor-based gene signature predicting prognosis of HBV/HCV-included resected hepatocellular carcinoma patients
Source: J Transl Med. 2019 Jun 18;17:203. doi: 10.1186/s12967-019-1946-8 (PMC6582497; doi:10.1186/s12967-019-1946-8)
Supplement: Supplementary file 1 — Additional file 1: Table S1. Name list of the genes tested by Nanostring in our study for further analysis. [file 12967_2019_1946_MOESM1_ESM.docx]

**Table S1.** Name list of the genes tested by Nanostring in our study for further analysis

ABTB1, ACE, ACKR3, ACOT9, ACTA2, ACTR2, ACTR3B, ACVR1B, ACVR1C, ACVR2A, ACVRL1, ACY1, ADAM10, ADAM9, ADAMDEC1, ADAMTS4, ADAP2, ADGRG5, ADH1A, ADH1B, AFP, AGFG2, AGTR1, AGTRAP, AHR, AIM2, AKR1B10, AKR1D1, AKT1, AKT2, AKT3, ALB, ALDH1A1, ALDH1A2, ALDH1L1, ALDH2, ALDH3A1, ALDH9A1, ALS2CL, AMPD1, ANGPT1, ANGPT2, ANGPTL4, ANLN, ANPEP, ANXA1, APCS, APLN, APOB, APOBEC3F, APOBEC3G, APOC3, ARAF, AREG, ARG1, ARHGAP15, ARHGAP31, ASCL2, ASZ1, ATF3, ATP6V1G2, AXIN1, AXIN2, BAD, BAG1, BAK1, BAMBI, BANK1, BATF, BAX, BBC3, BCL10, BCL2, BCL2A1, BCL2L1, BCL2L11, BCL3, BCL6, BEX1, BGN, BID, BIRC3, BIRC5, BLK, BLMH, BLNK, BLVRA, BMF, BMP2, BMP4, BMP5, BMP6, BMP7, BMP8A, BMPR1B, BMPR2, BRAF, BRD2, BRD3, BRD4, BRDT, BRIP1, BST1, BST2, BTK, BTLA, BTN3A3, C15orf48, C1orf116, C1orf162, C2CD2L, C5AR1, CACNA1C, CALD1, CALR, CAPN2, CAPN6, CARD11, CASP1, CASP3, CASP8, CBL, CCL18, CCL19, CCL2, CCL20, CCL21, CCL22, CCL28, CCL3, CCL4, CCL5, CCL8, CCNB1, CCND1, CCND2, CCNE1, CCNI, CCR3, CCR4, CCR5, CCR6, CCR7, CCRL2, CD14, CD160, CD163, CD163L1, CD19, CD1A, CD1C, CD1D, CD2, CD207, CD209, CD22, CD226, CD24, CD244, CD247, CD248, CD27, CD274, CD276, CD28, CD302, CD33, CD34, CD36, CD37, CD3D, CD3E, CD3G, CD4, CD40, CD40LG, CD44, CD47, CD48, CD6, CD68, CD69, CD7, CD70, CD72, CD79A, CD79B, CD80, CD83, CD86, CD8A, CD8B, CD96, CDC20, CDC42, CDC42EP3, CDC42SE2, CDC6, CDH1, CDH2, CDH3, CDH5, CDK4, CDK6, CDKN1A, CDKN2A, CDKN2B, CDX2, CEACAM1, CEACAM8, CEBPA, CEBPB, CELF2, CEMIP, CENPF, CENPK, CEP55, CFHR1, CFHR2, CHEK1, CHI3L1, CHST12, CIITA, CISH, CLC, CLEC10A, CLEC12A, CLEC4C, CLU, CMTM2, COL4A1, COL4A2, COL4A6, COL5A1, COL6A1, COL6A2, COL8A1, COPS3, CPNE5, CR1, CR2, CRABP2, CREBBP, CRK, CSDC2, CSF1, CSF1R, CSF2, CSF2RB, CSF3, CSF3R, CTAG1B, CTBP1, CTGF, CTLA4, CTNNA2, CTNNB1, CTSK, CX3CL1, CX3CR1, CXCL1, CXCL10, CXCL11, CXCL12, CXCL13, CXCL3, CXCL8, CXCL9, CXCR3, CXCR4, CXCR5, CXXC5, CYBB, CYP1A1, CYP1B1, CYP2B6, CYP2C18, CYP2C19, CYP2E1, CYP3A4, CYP3A7, CYTIP, DBF4, DCT, DDB1, DDC, DDR2, DENND2D, DENND3, DHFR, DKK1, DKK3, DLC1, DLK1, DLL1, DLL3, DLL4, DNMT3L, DNPH1, DPEP2, DPP4, DTL, DTX4, DUSP10, DUSP2, DUSP4, DUSP5, DUSP6, DUSP8, DVL1, DVL2, DVL3, E2F7, EAF2, Ebi3, ECT2, EDAR, EDNRA, EDNRB, EFNB1, EFNB2, EFS, EGFL7, EGFR, EGLN1, EGLN3, EHF, ENPP3, ENTPD1, EOMES, EP300, EPCAM, EPO, EPOR, EPS8, ERAP1, ERAP2, ERBB2, ERBB3, ERBB4, EREG, ERN1, ESM1, ESR1, ETS1, ETV6, EVI2A, EXO1, EXOSC9, EZH2, EZR, F2R, F3, FADS2, FAM129A, FAM129C, FAM189B, FAM20C, FAM26F, FAP, FAS, FASLG, FASN, FBN1, FBXO32, FBXO5, FBXW2, FBXW7, FCER1G, FCER2, FCGR1A, FCGR1B, FCGR1CP, FCGR2A, FCGR3A, FCN1, FCRL1, FCRL2, FCRL3, FCRL5, FCRLA, FERMT1, FGF1, FGF10, FGF11, FGF12, FGF13, FGF14, FGF16, FGF17, FGF18, FGF19, FGF2, FGF20, FGF21, FGF22, FGF23, FGF3, FGF4, FGF5, FGF6, FGF7, FGF8, FGF9, FGFBP2, FGFR1, FGFR2, FGFR3, FGFR4, FHL2, FLT1, FLVCR2, FN1, FOS, FOSB, FOSL1, FOSL2, FOXA1, FOXA2, FOXA3, FOXC1, FOXJ1, FOXM1, FOXO1, FOXO3, FOXP3, FPR1, FPR3, FRAT2, FSCN1, FSCN2, FST, FUT2, FUT4, FUT8, FYB, FYN, FZD1, FZD2, FZD7, FZD9, GAB1, GABBR1, GABRP, GALNT13, GAS1, GAS6, GAS7, GATA3, GATA4, GBP1, GBP5, GDF2, GEMIN5, GEMIN6, GGH, GIMAP5, GINS1, GLCCI1, GLI1, GLI2, GLI3, GLS, GLUL, GM2A, GNAS, GNLY, GNMT, GPC3, GPR160, GPR171, GPR174, GPR18, GRB2, GRB7, GSK3B, GUSB, GZMA, GZMB, GZMH, GZMK, H1F0, HABP2, HAS1, HAS2, HAS3, HAVCR2, HBEGF, HCK, HDAC5, HDAC9, HES1, HESX1, HEY1, HEY2, HEYL, HGF, HHEX, HHLA2, HIF1A, HIP1, HK3, HLA-C, HLA-DOB, HLA-DQA1, HMGB1, HMMR, HNF1A, HNF1B, HNF4A, HPSE, HRASLS, HS3ST3B1, HSP90AA1, HSPA13, HSPA6, IBSP, ICAM1, ICOS, IDO1, IFI27, IFI30, IFIT1, IFIT2, IFNG, IGF1R, IGF2, IGFBP3, IGFBP5, IGHM, IKBKE, IKZF2, IKZF3, IKZF4, IL10, IL10RB, IL12A, IL12b, IL12RB1, IL12RB2, IL13, IL16, IL17A, IL17B, IL17F, IL17RB, IL18, IL1a, IL1B, IL1RL1, IL1rn, IL2, IL21, IL21R, IL22, IL23A, IL23R, IL27, IL2RA, IL2RB, IL2RG, IL3, IL31, IL34, IL3RA, IL4, IL4R, IL5, IL6, IL6R, IL7, IL7R, IL9, INHBA, INHBE, INPP4B, IRF1, IRF3, IRF4, IRF7, IRF8, IRF9, IRS1, IRS2, ISG15, ITCH, ITGA2B, ITGA4, ITGA6, ITGAL, ITGAM, ITGAX, ITGB1, ITGB3, ITGB7, ITK, ITPKB, ITSN1, JAG1, JAG2, JAK1, JAK2, JAK3, JUN, JUNB, JUND, JUP, KAT5, KATNBL1, KCNJ2, KDELC1, KDM5A, KDM5B, KDR, KIAA0101, KIAA0125, KIF1A, KIF2C, KIR2DL1, KIR2DL3, KIR2DL4, KIR2DS2, KIR3DL1, KIT, KLF1, KLF6, KLHL14, KLRB1, KLRC1, KLRC2, KLRC3, KLRD1, KLRF1, KLRG1, KLRK1, KMO, KRAS, KRT14, KRT17, KRT19, KRT20, KRT5, KRT7, LAG3, LAIR1, LAMA3, LAMA4, LAMB1, LAMP1, LAPTM5, LCK, LCN2, LCP1, LCP2, LDHA, LDHB, LECT2, LEP, LEPR, LFNG, LGALS1, LGALS3, LGALS9, LGR4, LGR5, LGR6, LIF, LIFR, LILRA2, LILRB2, LILRB3, LMCD1, LMO2, LONP1, LRMP, LRP1, LRP4, LRP5, LRP6, LRRC32, LST1, LTA, LTF, Ly6E, LY75, LY9, LyPD1, MAFB, MAGEA3, MAGEA4, MAML1, MAML2, MAML3, MAP2K1, MAP2K2, MAP4K1, MAPK3, MAPK8, MAPRE1, MAPRE2, MAPT, MARCKS, MARCKSL1, MCAM, MDM2, MECP2, MED31, MED8, MELK, MET, MFAP2, MFAP5, MFNG, MGLL, MIA, MIB1, MICA, MICB, MITF, MKI67, MLANA, MLH1, MLPH, MME, MMP1, MMP11, MMP14, MMP16, MMP2, MMP7, MMP9, MPEG1, MPL, MRAS, MRC1, MS4A1, MS4A7, MSH2, MSH6, MSLN, MSR1, MST1R, MSX1, MTOR, MUC1, MUC16, MVP, MXRA8, MYBL1, MYBL2, MYC, MYL9, MYO1B, MYO5C, NAT1, NBL1, NCAM1, NCF1, NCF2, NCF4, NCOA4, NCOA7, NCR1, NCR3, NCR3LG1, NDC80, NDRG2, NECTIN2, NEK6, NELL1, NETO2, NF1, NFAM1, NFKB1, NFKB2, NID1, NID2, NIPSNAP1, NKD1, NKD2, NKG7, NLRC3, NLRP1, NLRP3, NNMT, NOD1, NOD2, NODAL, NOG, NOS2, NOTCH1, NOTCH2, NOTCH3, NOTCH4, NR1H4, NR4A1, NR5A2, NRARP, NREP, NRG1, NT5E, NUAK1, NUF2, OGT, OLFML2a, OLR1, ONECUT1, ORC6, OSBPL10, OSM, P2RX5, PAK1, PALLD, PATL2, PCOLCE, PDCD1, PDCD1LG2, PDE4A, PDGFRA, PDGFRB, PDLIM4, PDPN, PDX1, PDZK1IP1, PECAM1, PEG3, PGF, PGM1, PGR, PHB, PHC2, PHGDH, PHLPP1, PHLPP2, PI3, PIGF, PIK3CA, PIK3CB, PIK3CD, PIK3CG, PIK3R1, PIK3R2, PIK3R3, PIK3R5, PILRA, PIM1, PIM2, PIM3, PKIA, PLA2G5, PLA2G7, PLEKHO1, PLPP5, PMAIP1, PMEL, PMEPA1, PMS2, PNOC, POLE, POLK, POU2AF1, PPARG, PPP2R5A, PPP2R5C, PRAM1, PRAME, PRDX2, PRDX3, PRF1, PRKACA, PRKCH, PRKCQ, PRL, PRLR, PROCR, PROK2, PROM1, PROX1, PRSS1, PSMB8, PSMB9, PTAFR, PTCH1, PTEN, PTGDR, PTGER2, PTGER3, PTGER4, PTGES, PTGES2, PTGES3, PTGS1, PTGS2, PTPN1, PTPN22, PTPN7, PTPRC, PTPRCAP, PTPRE, PTTG1, PTX3, PVR, PVRIG, PYGL, PYHIN1, RAB2A, RABAC1, RAD23B, RAD51AP1, RAET1E, RAF1, RALGPS2, RARA, RARRES1, RARRES3, RASAL2, RASAL3, RASGRP4, RASL10A, RASSF2, RB1, RBBP9, RDX, RELN, RFNG, RHOBTB3, RIN1, RNF144B, RNF149, RNF43, RORa, RORC, RPS6KA1, RRM2, RSPO1, RSPO2, RSPO3, RSPO4, RUNX1, RUNX2, S100A1, S100A8, S100A9, S100B, SALL2, SAMD3, SAMSN1, SCAP, SCARB1, SCG3, SCGB2A2, SDC1, SDHA, SEC14L1, SELE, SELP, SEMA4A, SERPINA1, SERPINA5, SERPINA9, SERPINB1, SERPINE1, SERTAD1, SFRP1, SFRP2, SFRP4, SH3BP5, SIGLEC1, SIRPA, SLA, SLAMF7, SLAMF8, SLC15A3, SLC31A2, SLC34a2, SLC37A1, SLC39A6, SLC7A7, SMAD1, SMAD2, SMAD3, SMAD4, SMAD5, SMAD6, SMAD7, SMAD9, SMARCA4, SMARCD1, SMO, SMYD1, SNAP91, SOCS1, SOCS2, SOCS3, SOS1, SOS2, SOST, Sox17, SOX8, SOX9, SP2, SPATS2, SPI1, SPOCD1, SPP1, SPRY2, SPRY4, SQSTM1, SRPX2, SSH3, STAB1, STAP1, STAT1, STAT2, STAT3, STAT4, STAT5A, STAT5B, STAT6, STEAP1, STEAP3, STK11, STMN1, STT3A, SUFU, SULT1A1, SUOX, SUSD6, SYK, SYMPK, TAGLN, TAP1, TAP2, TAPBP, TBC1D27, TBP, TBX21, TBX3, TCF3, TCF7L1, TCL1A, TDO2, TFRC, TGFA, TGFB1, TGFB2, TGFB3, TGFBR1, TGFBR2, THBD, THBS1, THPO, THY1, TIFA, TIGIT, TIMD4, TIMP1, TKT, TLE3, TLR2, TLR3, TLR4, TLR5, TLR9, TMED2, TMEM176A, TMEM45B, TMEM55B, TNC, TNF, TNFAIP2, TNFAIP3, TNFAIP6, TNFRSF10A, TNFRSF10C, TNFRSF11A, TNFRSF13C, TNFRSF14, TNFRSF17, TNFRSF18, TNFRSF1B, TNFRSF4, TNFRSF9, TNFSF11, TNFSF14, TNFSF18, TNFSF4, TNFSF9, TOP1, TOP2A, TOX, TP53, TP63, TP73, TPST1, TRADD, TRAF1, TRAT1, TREM1, TREM2, TRIB2, TRIM21, TRIM69, TRMT6, TRO, TROVE2, TSC1, TSC2, TSPO, TTF1, TUBB, TWIST1, TXN2, TYMP, TYMS, UBASH3B, UBD, UBE2C, UBE2L6, UBE2T, UBN1, UCHL1, ULBP2, ULBP3, UNC5B, VAV2, VCAM1, VCAN, VEGFA, VEGFC, VIM, VNN2, VPREB3, VPS33B, VTCN1, WHSC1, WIPF1, WISP1, WNT1, WNT10B, WNT11, WNT3, WNT4, WNT5A, WNT9A, WT1, XBP1, XIST, ZAP70, ZBP1, ZC2HC1A, ZCCHC14, ZEB1, ZEB2, ZNF101, ZNF367, ZNF385A, ZNRF3,

Note: The genes whose raw data of expression levels did not meet the criteria of data

quality control were not shown in this list.
