# Supplementary material for: Development and validation of a new tumor-based gene signature predicting prognosis of HBV/HCV-included resected hepatocellular carcinoma patients
Source: J Transl Med. 2019 Jun 18;17:203. doi: 10.1186/s12967-019-1946-8 (PMC6582497; doi:10.1186/s12967-019-1946-8)
Supplement: Supplementary file 2 — Additional file 2: Table S2. The coefficient and regulation of 9-gene signature. [file 12967_2019_1946_MOESM2_ESM.docx]

**Table S2.** The coefficient and regulation of 9-gene signature.

| **Gene** | **Gene description** | **Coefficient** | **Regulation** |
| --- | --- | --- | --- |
| ZC2HC1A | zinc finger C2HC-type containing 1A | 0.142772 | Up |
| MARCKSL1 | MARCKS like 1 | 0.109389 | Up |
| PTGS1 | prostaglandin-endoperoxide synthase 1 | 0.097517 | Up |
| CDKN2B | cyclin dependent kinase inhibitor 2B | 0.001941 | Up |
| CLEC10A | C-type lectin domain family 10, member A | -0.00375 | Down |
| PRDX3 | peroxiredoxin 3 | -0.03609 | Down |
| PRKCH | protein kinase C eta | -0.06777 | Down |
| MPEG1 | macrophage expressed 1 | -0.07912 | Down |
| LMO2 | LIM domain only 2 | -0.14638 | Down |
