# Supplementary figures and images for: Development and validation of a new tumor-based gene signature predicting prognosis of HBV/HCV-included resected hepatocellular carcinoma patients
Source: J Transl Med. 2019 Jun 18;17:203. doi: 10.1186/s12967-019-1946-8 (PMC6582497; doi:10.1186/s12967-019-1946-8)

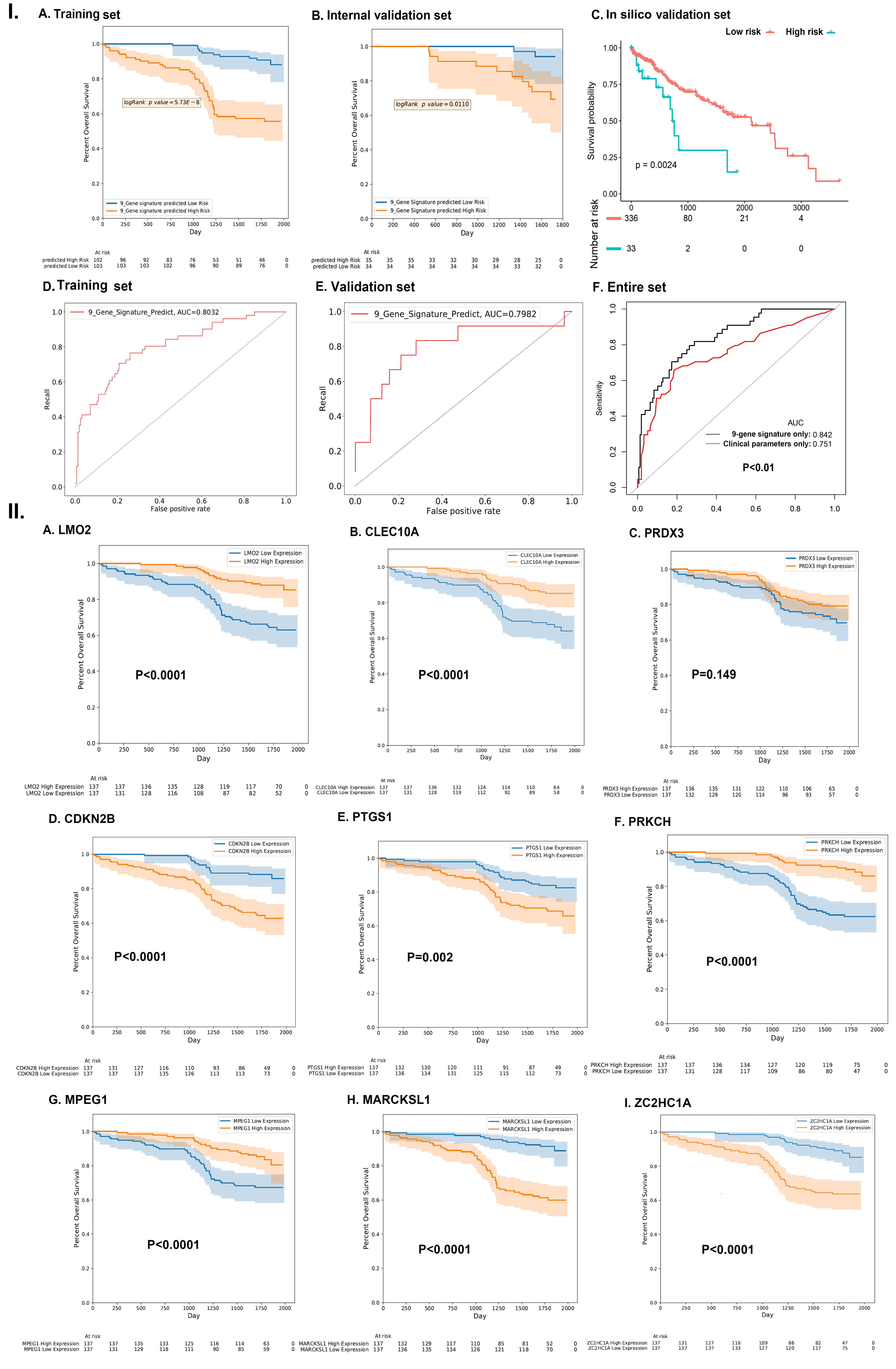

Supplement: Supplementary file 3 — Additional file 3: Figure S1. The area under the curve related to overall survival, the Kaplan–Meier analyses according to 9-gene signature (Panel I) and each nine genes (Panel II) in training and validation cohorts. Panel I: A: training set; B: internal validation set; C: in silico validation set; D. training set; E. validation set; F. entire set. Panel II: The genes were divided by median expression. [file 12967_2019_1946_MOESM3_ESM.tif]

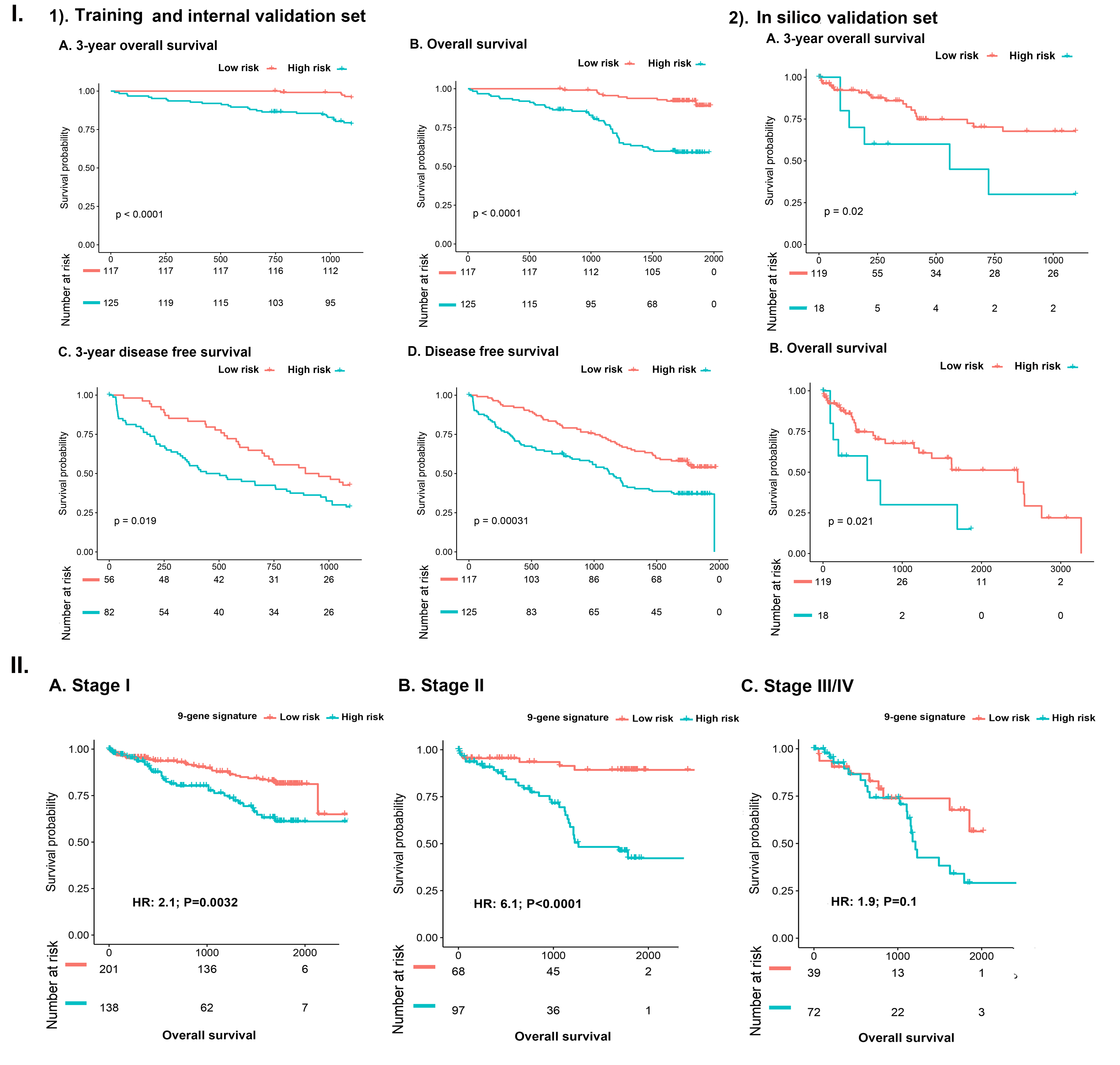

Supplement: Supplementary file 4 — Additional file 4: Figure S2. The time-dependent area under the curve related to overall survival, disease-free survival at different years (Panel I; 1-year, 3-year and 5-year) and the subgroup analysis for 9-gene signature in different clinical characteristics (Panel II). [file 12967_2019_1946_MOESM4_ESM.tif]

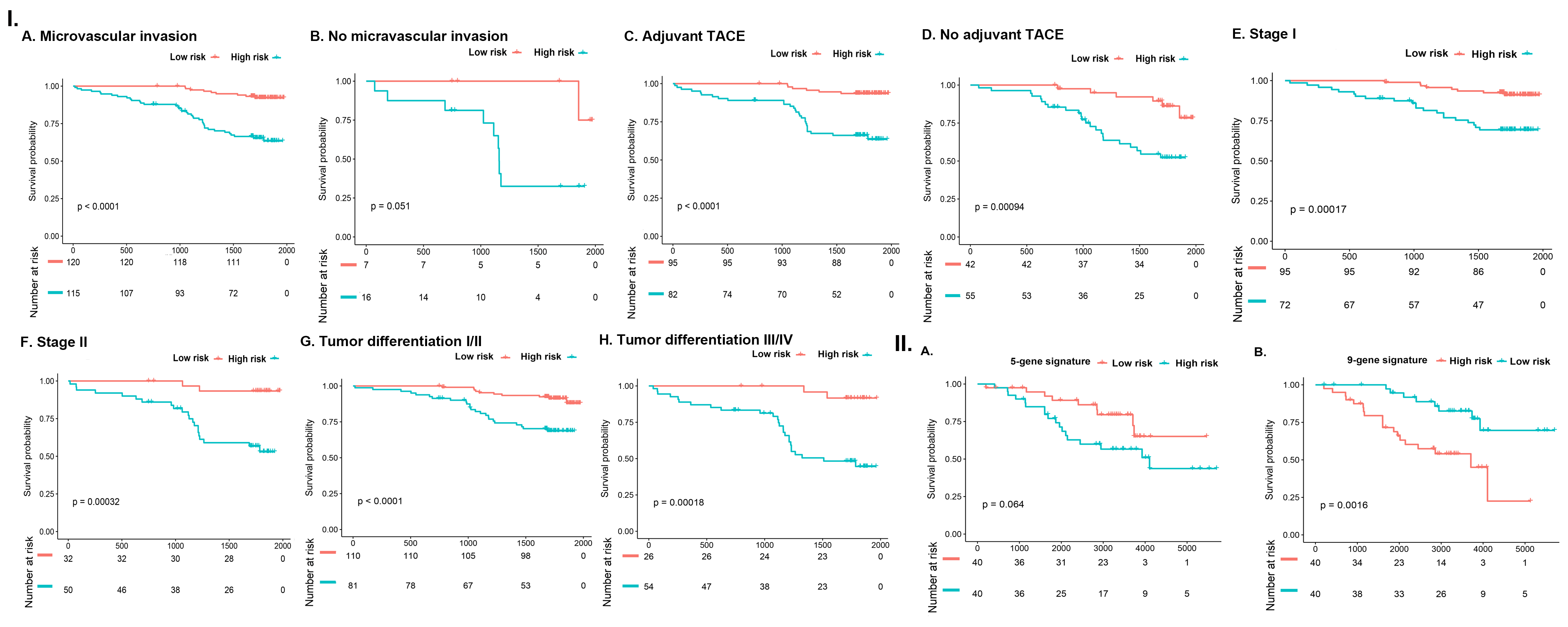

Supplement: Supplementary file 6 — Additional file 6: Figure S3. The Kaplan–Meier analyses according to 9-gene signature in different subgroups (Panel I) and the comparison of 9-gene signature with other molecular score for Kaplan–Meier analyses analysis. Panel II: A: 5-gene signature; B: 9-gene signature. [file 12967_2019_1946_MOESM6_ESM.tif]
