# Supplementary material for: Development and validation of a new tumor-based gene signature predicting prognosis of HBV/HCV-included resected hepatocellular carcinoma patients
Source: J Transl Med. 2019 Jun 18;17:203. doi: 10.1186/s12967-019-1946-8 (PMC6582497; doi:10.1186/s12967-019-1946-8)
Supplement: Supplementary file 5 — Additional file 5: Table S3. Correlation between 9-gene signature and clinicopathologic characteristics from training and validation cohort. [file 12967_2019_1946_MOESM5_ESM.docx]

**Table S3.** Correlation between 9-gene signature and clinicopathologic characteristics from training and validation cohort.

|  | 9-gene signature predict | |  |
| --- | --- | --- | --- |
| Characteristics | Low risk^*^ | High risk^*^ | P value |
| Age |  |  | 0.873 |
| <60 | 177 (49.72%) | 179 (50.28%) |  |
| >=60 | 144 (50.35%) | 142 (49.65%) |  |
| Gender |  |  | 0.429 |
| Female | 78 (47.27%) | 87 (52.73%) |  |
| Male | 243 (50.84%) | 235 (49.16%) |  |
| HBV |  |  | 0.172 |
| Negative | 33 (61.11%) | 21 (38.89%) |  |
| Positive | 194 (51.19%) | 185 (48.81%) |  |
| Adjuvant TACE |  |  | 0.245 |
| No | 224 (55.31%) | 181 (44.69%) |  |
| Yes | 54 (49.09%) | 56 (50.91%) |  |
| Microvascular invasion |  |  | **0.021** |
| No | 245 (55.30%) | 198 (44.70%) |  |
| Yes | 56 (43.75%) | 72 (56.25%) |  |
| Tumor number |  |  | 0.068 |
| Single | 186 (57.94%) | 135 (42.06%) |  |
| Multiple | 61 (48.41%) | 65 (51.59%) |  |
| Tumor diameter (cm) |  |  | **<0.001** |
| <5 | 87 (75.00%) | 29 (25.00%) |  |
| >=5 | 39 (45.35%) | 47 (54.65%) |  |
| Stage |  |  | **<0.001** |
| I | 201 (64.8%) | 138 (44.5%) |  |
| II | 68 (21.9%) | 97 (31.3%) |  |
| III/IV | 41 (13.2%) | 75 (24.2%) |  |
| Tumor differentiation |  |  | **<0.001** |
| I | 159 (64.90%) | 86 (35.10%) |  |
| II/III | 160 (41.03%) | 230 (58.97%) |  |
| AFP (ng/ml) |  |  | 0.132 |
| <200 | 195 (55.24%) | 158 (44.76%) |  |
| >=200 | 84 (48.28%) | 90 (51.72%) |  |
| Note: ^*^ Low or high risk was divided by gene median expression; TACE: transarterial chemoembolization; AFP: alpha-fetoprotein. | | | |
