# Supplementary material for: Development and validation of a new tumor-based gene signature predicting prognosis of HBV/HCV-included resected hepatocellular carcinoma patients
Source: J Transl Med. 2019 Jun 18;17:203. doi: 10.1186/s12967-019-1946-8 (PMC6582497; doi:10.1186/s12967-019-1946-8)
Supplement: Supplementary file 8 — Additional file 8: Table S4. Multivariable Cox regression of overall survival for patients with HBV-DNA positive tumors. [file 12967_2019_1946_MOESM8_ESM.docx]

**Table S4.** Multivariable Cox regression of overall survival for patients with HBV-DNA positive tumors.

| Parameter | HR (95% CI) | P value | ci Training  (95% CI) | ci Validation  (95% CI) | |
| --- | --- | --- | --- | --- | --- |
| 9-gene signature | 6.05 (3.77, 9.72) | <0.0001 | 0.77 (0.65, 0.89) | 0.73 (0.55, 0.85) | |
| Clinical parameters | | | | |  |
| Tumor diameter | 1.17(1.09, 1.24) | 0.0001 |  |  | |
| Tumor differentiation | 2.08 (1.01, 4.39) | 0.0003 | 0.68 (0.31, 1.04) | 0.69 (0.49, 0.89) | |
| **9-gene signature and clinical parameters** | | | | |  |
| 9-gene signature | 15.35 (5.53, 42.60) | <0.0001 |  |  | |
| Tumor diameter | 1.11 (1.03, 1.20) | 0.007 |  |  | |
| Tumor differentiation | 1.55 (0.64, 3.78) | 0.337 | 0.85 (0.75, 0.95) | 0.82 (0.68, 0.96) | |
| **Improvement of combined model compared to** |  | dLL | Degrees of freedom | P | |
| 9-Gene signature only |  | 5.68 | 2 | 0.01 | |
| Clinical parameters only |  | 12.21 | 2 | 0.001 | |

Note: Three multivariable Cox regression models were built using the training cohort: a model consisting of only the 9-gene signature (top), a model consisting only of the clinical tumor diameter and tumor differentiation, and a model combining both the 9-gene signature and clinical parameters (bottom). HRs are given with their 95% CIs and the corresponding P values. For each model, the concordance index (ci) is given for the training and internal validation cohort as well as for the patients of the or in silico validation cohort. Its 95% CI is determined from 1,000 bootstrap samples of the respective cohort. The improvement of the combined model, including the 9-gene signature and the clinical parameters, compared with the 9-gene signature and clinical parameters alone is shown (bottom) based on the difference in log-likelihood (dLL).
