# Supplementary material for: Development and validation of a new tumor-based gene signature predicting prognosis of HBV/HCV-included resected hepatocellular carcinoma patients
Source: J Transl Med. 2019 Jun 18;17:203. doi: 10.1186/s12967-019-1946-8 (PMC6582497; doi:10.1186/s12967-019-1946-8)
Supplement: Supplementary file 9 — Additional file 9: Table S5. Comparison of the 9-gene signature and the 5-genes signature to predict overall survival using bivariate analysis in GEO set of patients (n=80). [file 12967_2019_1946_MOESM9_ESM.docx]

**Table S5.** Comparison of the 9-gene signature and the 5-genes signature to predict overall survival using bivariate analysis in GEO set of patients (n=80)

| Variables | HR (95%CI) | Wald test P value |
| --- | --- | --- |
| 9-gene signature^*^ |  |  |
| Low risk | 1.0 |  |
| High risk | 3.3 (1.7, 10) | 0.003 |
| 5-gene signature* |  |  |
| Low risk | 1.0 |  |
| High risk | 2.1 (0.9, 4.7) | 0.070 |

Note: ^*^low or high risk of gene signature was divided by median value
